# *Mycobacterium tuberculosis* PPE18 Protein Bodies in Insect Cells: A Candidate Tuberculosis Vaccine

**DOI:** 10.3390/vaccines13070671

**Published:** 2025-06-23

**Authors:** Pu Wang, Gang Zhang, Yurong Cai, Lingling Jiang, Xiaoxia Niu, Sinong Zhang, Weifeng Gao, Zhiwei Wu, Yong Li

**Affiliations:** 1 School of Life Sciences, Ningxia University, Yinchuan 750021, China; wangpu1175@163.com (P.W.); fuzang123@163.com (G.Z.); carits.cc@gmail.com (Y.C.); jianglingling0512@163.com (L.J.); 18695466964@163.com (X.N.); sinongzhang@nxu.edu.cn (S.Z.); stephanie.wf@163.com (W.G.); 2 Key Laboratory of Ministry of Education for Protection and Utilization of Special Biological Resources in Western China, Yinchuan 750021, China; 3 People’s Hospital of Ningxia Hui Autonomous Region, Ningxia Medical University, Yinchuan 750002, China; 4 Yunnan Provincial Key Laboratory of Entomological Biopharmaceutical R&D, College of Pharmacy, Dali University, Dali 671003, China; 5 Center for Public Health Research, Medical School, Nanjing University, Nanjing 210023, China

**Keywords:** PPE18, *Mycobacterium tuberculosis*, Zera nanoparticles, vaccine, immunity

## Abstract

Background/Objectives: *Mycobacterium tuberculosis* is the causative agent of tuberculosis and the leading cause of death from a single infection with the microorganism. Tuberculosis remains globally one of the major diseases leading to high mortality rates, with serious implications for public health and economic development. Therefore, tuberculosis prevention and control is crucial for global health and socio-economic stability. The development of effective preventive vaccines remains an urgent task in the fight against tuberculosis. Methods: The *Mycobacterium tuberculosis* antigen PPE18 was fused to Zera, and Bacmid was extracted and transfected into Sf9, which was purified and characterized for the formation of nanoparticle protein bodies. BALB/c mice and calves were immunized, and the immunogenicity of the nanoparticle vaccine was assessed by serum antibodies and splenic lymphocytes. Results: Zera-71CA-mCherry can be expressed in Sf9 cells, forming 0.5–1.2 μm protein bodies. Excising the mCherry sequence, Zera-71CA/Zera-PPE18 candidate nanoparticle-immunized mice were able to elicit serum antibody levels and the proliferation of splenic lymphocytes, and immunized calves were determined to have high levels of serum antibody levels, and IFN-γ and TNF-α levels. Conclusions: The results indicated that Zera-71CA/Zera-PPE18 recombinant nanoparticles had good immunogenicity as a subunit vaccine in both BALB/c mice and calves and are potential candidates for further development as effective subunit vaccines.

## 1. Introduction

Although the Bacillus Calmette–Guérin (BCG) vaccine has prevented millions of deaths over the past century, it has not been sufficient to substantially reduce the global burden of tuberculosis (TB). The BCG vaccine, while lifesaving, has not significantly reduced TB incidence, necessitating the development of a more effective vaccine to combat the persistent spreading of this deadly disease. Although several TB vaccines are currently in different clinical trial stages, they have not shown the expected protection against TB [1,2], which may be related to the insufficient immunogenicity of the *Mycobacterium tuberculosis* (*M. tb*) antigen.

The PE/PPE protein family, unique to pathogenic *Mycobacterium* [3,4], TB is defined by the presence of proline-glutamate (PE) and proline-proline-glutamate (PPE) motifs near their highly conserved N termini and plays a crucial role as virulence factors and T-cell antigens in *M. tb* [5]. PPE18 has been demonstrated to impair CD4⁺ T cell activation by inhibiting MHC class II-mediated antigen presentation in macrophages in a dose-dependent manner, while leaving the surface expression of MHC class II and costimulatory molecules unaffected. Additionally, mice infected with *M. tb* strains expressing PPE18 showed diminished B cell maturation and activation, accompanied by decreased levels of Mycobacteria-specific antibodies [6]. These findings suggest that PPE18 may serve as a mechanism by which *M. tb* evades adaptive immune responses, highlighting its potential as a target for antigen-based strategies aimed at improving the host defense against tuberculosis.

Zera-sequence-based nanoparticle delivery systems hold great potential for enhancing vaccine efficacy by inducing the formation of protein bodies (PBs) [7], which are organelles derived from the endoplasmic reticulum (ER) in maize seeds capable of stably storing large amounts of maize proteins. When alcohol-soluble maize protein polypeptides fused with the Zera sequence are expressed and directed to the ER for post-translational modification, they oligomerize and self-assemble into nanoscale PBs that can facilitate adaptive immune responses. These PBs can also be successfully formed in a wide range of eukaryotic cells, including *Nicotiana benthamiana* [8], insect Sf9 cells [9], mouse cells, Chinese hamster ovary (CHO) cells, and HEK293T cells [10,11], all demonstrating favorable immunogenicity. Notably, van Zyl A.R. and colleagues employed the Zera sequence expression system to develop a PB-based nanoparticle vaccine candidate against the bluetongue virus, which elicited a strong anti-VP2 immune response in mice without the need for adjuvants and required only minimal antigen doses [12]. Therefore, the Zera sequence offers a highly efficient and versatile platform for recombinant protein production by promoting their accumulation within ER-derived organelles, and the ability of PBs to form stable intracellular structures presents promising applications for non-secretory recombinant protein expression [13].

Based on the above properties of the Zera nanoparticle, we used the Zera nanoparticle delivery system to express the nanoparticles formed by the *M. tb* PPE18 protein and the MTB32C-PPE18 chimeric protein (referred to as Zera-71CA) as a subunit vaccine, and evaluated its immunogenicity in immunized mice and calves. The results showed that Zera-71CA could induce both humoral and cellular immune responses in mice and calves, suggesting that Zera-71CA is a potential candidate vaccine for TB; these results have laid the foundation for the future development of a subunit vaccine for TB prevention.

## 2. Materials and Methods

### 2.1. Design and Assembly of Expression Vectors Based on Selected Coding Sequences

Drawing on prior research on the PPE18 protein, we selected the gene sequence encoding the specific antigenic epitope of PPE18 from the *M. tb* H37Rv reference strain (GenBank ID: NC_000962.3). For MTB32A (GenBank ID: MTCI418B.07), a 396 bp C-terminal fragment—designated MTB32C—was isolated. We then designed coding sequences for both PPE18 and MTB32C to construct a fusion protein (MTB32C-PPE18) tagged with a C-terminal His tag, hereafter referred to as 71CA. To enable fluorescence detection, mCherry was incorporated as a reporter protein, and the Zera domain (GenBank: KU593570.1) was fused to the N-terminus of the target protein (Table 1). The complete construct was inserted under the control of the pH promoter in the pFastBacDual vector, yielding pFastBacDual-71CA-mCherry.

### 2.2. Cultivation of Sf9 Insect Cells and Production of Recombinant Zera-Based Nanoparticles

Sf9 cells (stored in our laboratory), maintained in our laboratory, were cultured at 27 °C in Sf-900 II serum-free medium (SFM) (Gibco, Grand Island, NY, USA). Zera-71CA-mCherry was cloned and inserted into the pFastBac dual vector (Invitrogen, Carlsbad, CA, USA) using the *Bam*HI and *Hin*dIII nucleic acid restriction sites (NEB, Beijing, China), and the obtained recombinant plasmids and pFastBac dual (negative control) were subsequently transformed into DH10Bac *E. coli* cells (Biomed). Positive clones were screened using blue–white selection and verified by sequencing, with the resulting constructs named Bacmid-Zera-71CA-mCherry and Bacmid-Dual, respectively. These bacmids were transfected into Sf9 cells using TransIT^®^-LT1 Transfection Reagent (Mirus) [14]. Following the manufacturer’s protocol, the fluorescence was observed at 72, 96, and 120 h post-transfection using a Nikon Eclipse TE2000-U inverted fluorescence microscope (Tokyo, Japan).

Infected Sf9 insect cells were lysed on ice for 20 min in lysis buffer (20 mM Tris, pH 7.5; 150 mM NaCl; 1% Triton X-100; and 10 μM PMSF), followed by centrifugation at 12,000× *g* for 10 min. The supernatant was transferred to PBP5 buffer (10 mM HEPES, pH 7.4; 2 mM EDTA) containing 10% sucrose and 10 μM PMSF, and then layered onto a discontinuous sucrose gradient (1.08, 1.13, 1.18, and 1.26 g/cm^3^). The gradient was centrifuged at 80,000× *g* for 2 h at 4 °C using a P40ST horizontal rotor (Himac, Tokyo, Japan). Fractions between 1.13–1.18 g/cm^3^ were collected to obtain the Zera-71CA/Zera-PPE18 nanoparticles.

### 2.3. Immunofluorescence Staining and Ultrastructural Analysis Using Transmission Electron Microscopy

To observe the subcellular localization of the fusion protein Zera-71CA-mCherry in infected Sf9 cells, cells were infected with Zera-71CA-mCherry recombinant nanoparticles at a multiplicity of infection (MOI) of 0.5. After infection, cells were fixed on slides using 4% paraformaldehyde and subsequently blocked with 5% bovine serum albumin for 2 h. Mouse anti-His monoclonal antibodies (Abcam, Shanghai, China, 1:500 dilution) were then applied and incubated overnight at 4 °C. Following PBS washes to remove unbound primary antibodies, CoraLite 488-conjugated goat anti-mouse IgG secondary antibody (Proteintech, Wuhan, China, 1:500 dilution) was added and incubated for an additional 2 h. After washing with PBS, DAPI was added dropwise and incubated at room temperature for 10 min. Fluorescence imaging was performed using a laser confocal microscope (Leica TCS SPE, Wetzlar, Germany). To further determine the subcellular localization of the fusion protein, Zera-71CA-mCherry, cells were examined using a transmission electron microscope operated at 80 kV (Hitachi H-7650, Tokyo, Japan).

### 2.4. Western Blot Identification of Vectors and Nanoparticles for Animal Immunoassays

To obtain proteins lacking fluorescent tags and the MTB32C sequence, two recombinant plasmids were constructed: pFastBac dual-Zera-71CA (with the mCherry tag removed) and pFastBac dual-Zera-PPE18 (with both the mCherry and MTB32C sequences removed). Recombinant Zera-71CA and Zera-PPE18 nanoparticles were then produced using the same protocol described previously. These nanoparticles were identified via Western blotting using mouse anti-His monoclonal antibody (Abcam, 1:500 dilution) and visualization with HRP-conjugated AffiniPure goat anti-mouse IgG (H+L) antibody (Proteintech, 1:5000 dilution).

### 2.5. Mouse Vaccination

A total of seventy-five healthy female BALB/c mice, aged between 6 and 8 weeks, were procured from Beijing Vitonda Biotechnology Co., Ltd. (Beijing, China, Certificate of Conformity No. SCXK Jing 2021-0006). These animals were randomly divided into five experimental groups, with each group comprising 15 mice, as detailed in Table 2. The immunization regimen involved the subcutaneous administration of vaccine formulations at biweekly intervals (14 days apart). The dosage of the commercial vaccines was determined based on established protocols reported in previously published studies [15,16]. Peripheral blood was collected from the retro-orbital sinus at multiple time points, specifically on days 0, 14, 28, 35, and 42 following the initial immunization, in order to evaluate the dynamics of the humoral immune response. Additionally, to assess the cellular immune response, three mice from each group were humanely euthanized by cervical dislocation at both day 35 and day 42 post-immunization. The spleens were aseptically excised and processed for lymphocyte proliferation assays. All experimental procedures involving animals were strictly conducted in accordance with the institutional guidelines and ethical standards. This study protocol received approval from the Experimental Animal Ethics Committee of Ningxia University (Ethics Approval No. NXU-2023-026), ensuring compliance with national regulations governing the care and use of laboratory animals.

### 2.6. IgG Antibody Analysis

IgG antibody levels were measured by indirect ELISA. Microtiter plates were coated with the protein antigen PPE18 (1 µg/mL, stored at −80 °C in the laboratory), followed by incubation with serum samples diluted 1:100, collected at 0, 14, 28, 35, and 42 days after immunization (DAI). Horseradish peroxidase (HRP)-conjugated goat anti-mouse IgG (Proteintech, 1:1000 dilution) was used as the secondary antibody. Each sample was tested in triplicate, and the absorbance was recorded at 450 nm using a microplate reader (EnSpire, Austin, TX, USA).

### 2.7. Lymphocyte Proliferation Assay

Mice from each group at 35 and 42 days after immunization were killed by neck-breaking, and the spleens were aseptically removed and ground using splenic lymphocyte isolation solution (Dakewe, Shenzhen, China), centrifuged at 800× *g* with a horizontal rotor for 30 min, and the cells were aggregated to form a white membrane layer with a clear interface. The cells in the white membrane layer were aspirated into 10 mL of RPMI 1640 (Gibco, Waltham, MA, USA) and centrifuged at 250× *g* for 10 min to collect the cells. Ten micrograms of Zera-71CA or Zera-PPE18 in 100 μL of RPMI 1640 (Gibco) were used as stimulants, and RPMI 1640 and knife-bean protein A (5 μg/mL; Solarbio, Beijing, China) were added as negative and positive controls, respectively. Following 42 h of stimulation, splenic lymphocyte proliferation was assessed using the MTT assay (5 mg/mL; Solarbio). The optical density (OD) was measured at 490 nm, and the stimulation index was calculated using the following formula: stimulation (%) = the mean OD of antigen-stimulated cells (Zera-71CA or Zera-PPE18)/the mean OD of unstimulated control cells (RPMI 1640).

### 2.8. Flow Cytometry

Forty-two days after immunization, the spleens of the mice were collected in a sterile environment, and the mouse splenic lymphocytes (Dakewe) were isolated and inoculated in 6-well plates with 1 × 10^6^ mouse splenocytes per well and stimulated with 5 μg/mL of Zera-71CA/Zera-PPE18 and 100 μL of PBS for 12 h. For the staining of the cell surface, the following antibodies (Proteintech) were used: CoraLite^®^Plus 405 Anti-Mouse CD4 (GK1.5), PE Anti-Mouse CD3 (17A2), and CoraLite^®^Plus 705 Anti-Mouse CD8a (53-6.7). Subsequently, the cells were fixed, permeabilized, and stained for intracellular cytokines using CoraLite^®^Plus 647-conjugated Anti-Mouse IFN-γ (XMG1.2) antibodies. The stained cells were then analyzed using a SONY MA900 flow cytometer, and the data were processed with FlowJo version 10 software (Tree Star Inc., Ashland, OR, USA).

### 2.9. Safety Assessment

To assess vaccine safety, mice were euthanized two weeks after the final immunization. Major organs were harvested, fixed in 4% paraformaldehyde, and submitted to Wuhan Servicebio for histopathological analysis.

### 2.10. Vaccination Strategies and Cytokine Detection in Calves

Fifteen 21-day-old Holstein calves were randomly assigned to five groups, with three calves per group. The calves were initially subcutaneously immunized with the Zera-71CA/Zera-PPE18 nanoparticle vaccine in the neck, and the commercial vaccine dosage was determined based on previous studies [17]. Blood samples were collected intravenously at 30, 60, and 90 days post-immunization, and the serum was separated to measure the levels of the cytokines TNF-α and IFN-γ. All calf experiments were performed in accordance with the guidelines of the Experimental Ethics Committee of the Ningxia University Laboratory Animal Center.

### 2.11. Statistical Analysis

Statistical analysis was conducted using GraphPad Prism^®^ version 6 for Windows (GraphPad Software, San Diego, CA, USA), with data presented as the mean ± SD. One-way analysis of variance (ANOVA) was employed to compare immune responses between groups. The results were considered statistically significant if *p* < 0.05 (*), *p* < 0.01 (**), or *p* < 0.001 (***), and “ns” indicates no statistical difference.

## 3. Results

### 3.1. Construction and Characterization of the PPE18 Expression Vector

**Figure 1:**
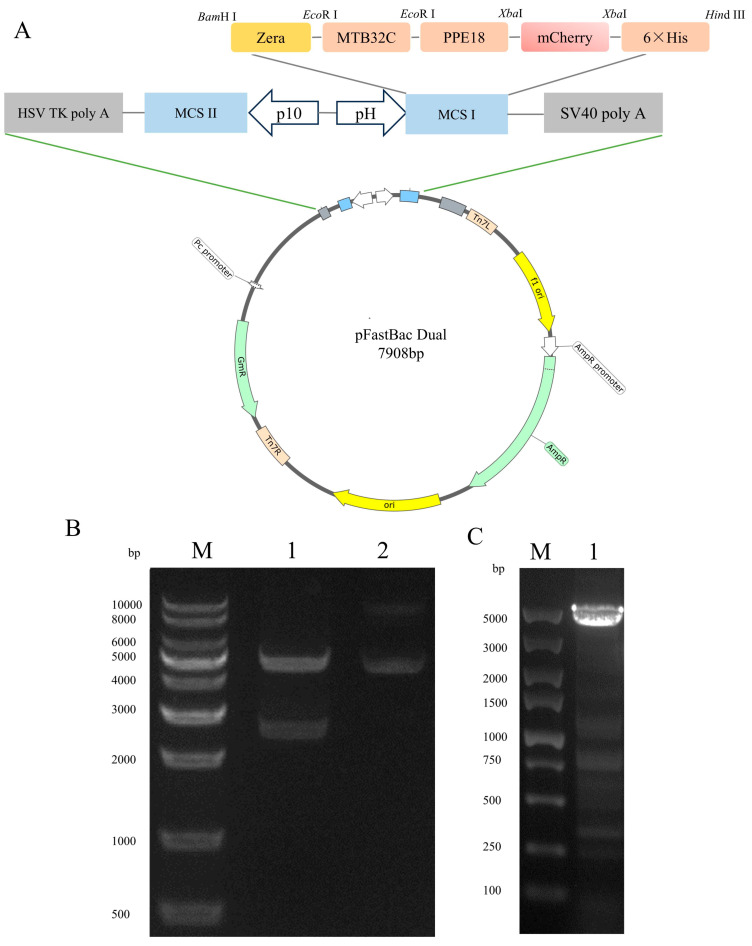
(**A**) Profile of the recombinant plasmid pFastBac dual-Zera-71CA-mCherry. (**B**) Double digestion of the recombinant plasmid pFastBac dual-Zera-71CA-mCherry. Lan M is DNA Marker, Lane 1 shows the *Bam*HI-*Hin*dII double-digested product of pFastBac dual-Zera-71CA-mCherry (5238 bp–2670 bp), while Lane 2 displays the recombinant vector plasmid. (**C**) PCR identification of the recombinant nanoparticle Bacmid-Zera-71CA-mCherry. Lan M is DNA Marker, Lane 1 is Bacmid-Zera-71CA-mCherry (5230 bp).

The recombinant plasmid designated pFastBac Dual-Zera-MTB32C-PPE18-mCherry (abbreviated as pFastBac Dual-Zera-71CA-mCherry; see Figure 1A) was successfully constructed through gene synthesis of the MTB32C and PPE18 sequences. These synthesized genes were subsequently cloned into the vector using a double restriction enzyme digestion strategy, the results of which are illustrated in Figure 1B. The accuracy of the gene insertion and orientation was further confirmed through DNA sequencing analysis. To verify that the target gene was successfully integrated into the bacmid, colony PCR amplification was performed using the universal M13 primer. The appearance of the expected amplification product indicated a correct construction (Figure 1C), and the recombinant bacmid was named Bacmid-Zera-71CA-mCherry.

### 3.2. Localization of the Recombinant Nanoparticle Zera-71CA-mCherry Antigen

**Figure 2:**
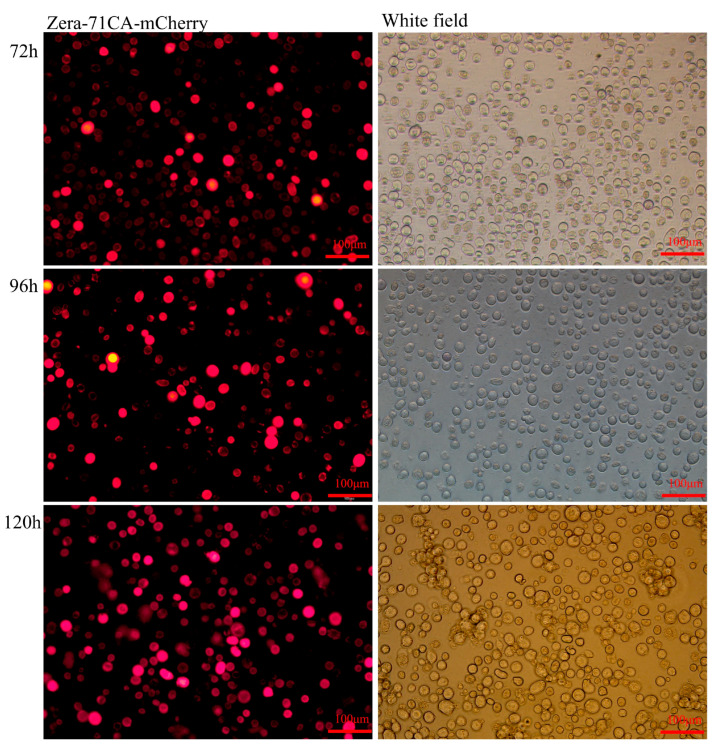
The mCherry fluorescent protein was used to visualize Zera-71CA-mCherry-infected Sf9 cells.

**Figure 3:**
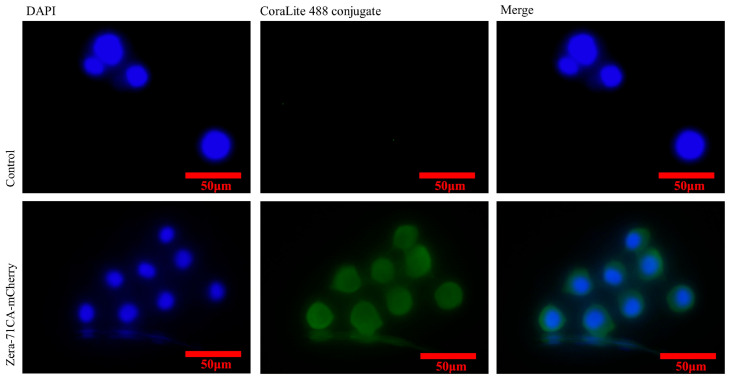
Indirect immunofluorescence analysis of the recombinant Zera nanoparticles and cells infected with the wild-type virus.

**Figure 4:**
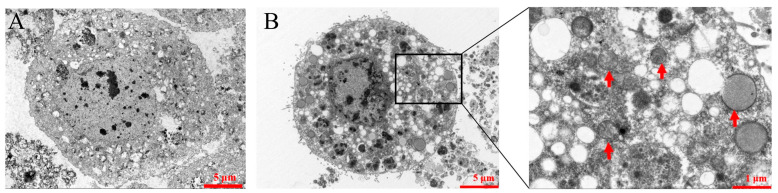
Electron micrographs of recombinant Zera nanoparticles. (**A**) Electron micrograph of uninfected SF9 cells. (**B**) Electron micrograph of recombinant Zera-71CA-mCherry nanoparticles; red arrows indicate formed Zera nanoparticles.

Sf9 cells transfected with Zera-71CA-mCherry were examined at 72, 96, and 120 h with different fields of view (white, red) and positive cells were identified by indirect immunofluorescence. The results showed that the antigen was localized to the plasma membrane of the infected Sf9 cells or highly enriched in its vicinity (Figure 2 and Figure 3). To determine whether the antigen could trigger PB formation, Zera-71CA-mCherry nanoparticles were examined by electron microscopy, as shown in Figure 4. The presence of abundant electron-dense organelle PBs was observed in Sf9 cells, and these dense vesicles were of different sizes (0.5–1.2 μm).

### 3.3. Zera-71CA and Zera-PPE18 Nanoparticles Induce Immune Responses in Mice

**Figure 5:**
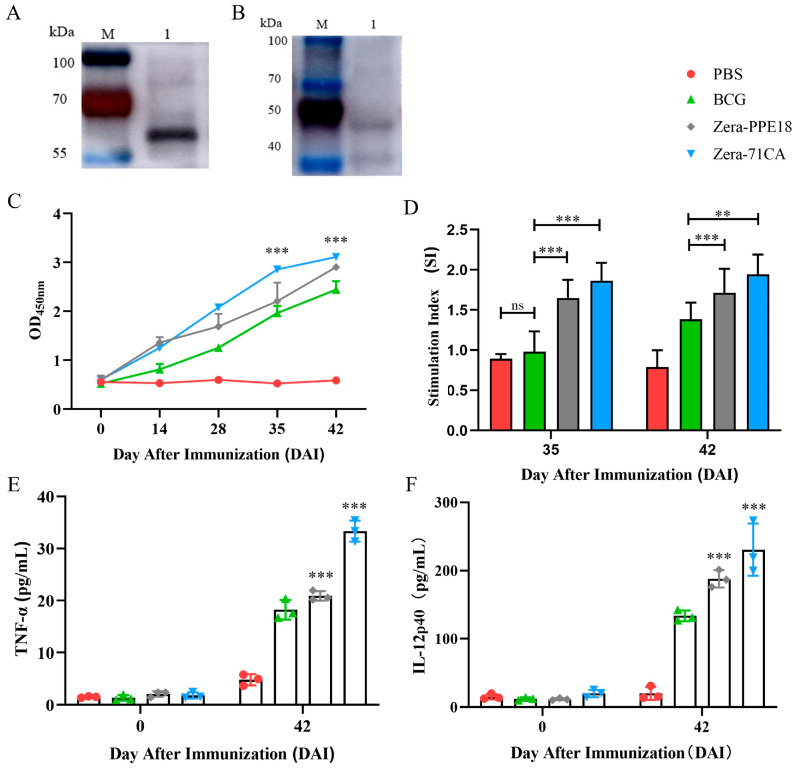
Western blot and immunogenicity assessment of the recombinant Zera nanoparticles in mice. (**A**) Western blotting of the recombinant Zera nanoparticle Zera-71CA (65.8 kDa). (**B**) Western blotting of the recombinant Zera nanoparticle Zera-PPE18 (52.8 kDa). (**C**) Indirect ELISA showing the immune response induced by recombinant Zera nanoparticles in mice. The y-axis represents the optical density (OD) at 450 nm for the serum samples collected at 0, 14, 28, 35, and 42 days after immunization (DAI) for each group. (**D**) Lymphocyte proliferation assay results. The y-axis indicates the stimulation index of splenic lymphocytes collected at 35 and 42 DAI. ns: not significant; ** *p* < 0.01, *** *p* < 0.001, indicating statistically significant differences (one-way ANOVA with Bonferroni correction). Quantification of the TNF-α (**E**) and IL-12p40 (**F**) levels in the sera of immunized mice. All experiments were performed in triplicate, and error bars represent the standard deviation (SD) (n = 3).

Two different Zera fusion protein nanoparticles, named Zera-71CA and Zera-PPE18, were successfully constructed by enzymatically ligating the coding sequences of the fluorescent proteins and/or MTB32C antigens in the recombinant plasmids. The aim of the above constructs was to explore the effects of different antigenic components on the immune response and to realize efficient expression and self-assembly based on the Zera platform. To verify the expression of the two recombinant proteins, Western blotting was performed using the anti-6XHis tag antibody. The results showed that clear specific bands were visible at the corresponding molecular weights (Figure 5A,B), indicating that the Zera-71CA and Zera-PPE18 fusion proteins were successfully synthesized in the expression system and the purified samples had good antigenicity. To further evaluate the humoral immune effects induced by these nanovaccine candidates in vivo, mouse sera were collected at different time points and IgG levels were detected by indirect enzyme-linked immunosorbent assay (ELISA) (Figure 5C). The experimental results showed that the antigen-specific IgG antibody titers in the sera of mice in both the Zera-71CA and Zera-PPE18 groups were significantly higher than those in the negative control and BCG-immunized groups on the 35th and 42nd days after immunization, and the results of the statistical analyses were highly significant (*p* < 0.001). These results suggested that the Zera nanoparticle vaccine can effectively stimulate a strong specific humoral immune response in mice.

In order to comprehensively assess the ability of Zera-71CA and Zera-PPE18 nanoparticle vaccines to induce cellular immune response, this study examined the proliferative response of splenic lymphocytes in mice on day 35 and 42 after immunization, respectively. The results showed that the stimulation index (SI) of these two groups of mice was significantly higher than that of the negative control group (PBS) and the positive control group (BCG-immunized group), and the difference was highly statistically significant (Figure 5D). To further explore the cytokine profile of the cellular immune response elicited by the nanovaccine, the study was conducted by collecting mouse serum on day 42 after immunization and determining the expression levels of the pro-inflammatory cytokines IL-12p40 and TNF-α. The data showed that the mean concentrations of IL-12p40 and TNF-α reached 209.38 ± 13.84 pg/mL and 34.34 ± 1.49 pg/mL, respectively, in the Zera-71CA-immunized group, whereas in the Zera-PPE18 group, the levels of IL-12p40 and TNF-α were 181.25 ± 7.66 pg/mL and 21.09 ± 1.23 pg/mL. Compared with the PBS control and BCG-immunized groups, the cytokine expression levels of both Zera nanovaccine groups were significantly elevated, and the differences were all statistically significant (*p* < 0.001, Figure 5E,F). These results indicated that the nanoparticle vaccines constructed on the Zera platform could significantly promote the secretion of Th1-type immune-related factors and possessed good potential for cellular immunity induction.

### 3.4. The Zera-71CA/Zera-PPE18 Nanoparticle Vaccine Activates T-Cell Immune Response in Mice

**Figure 6:**
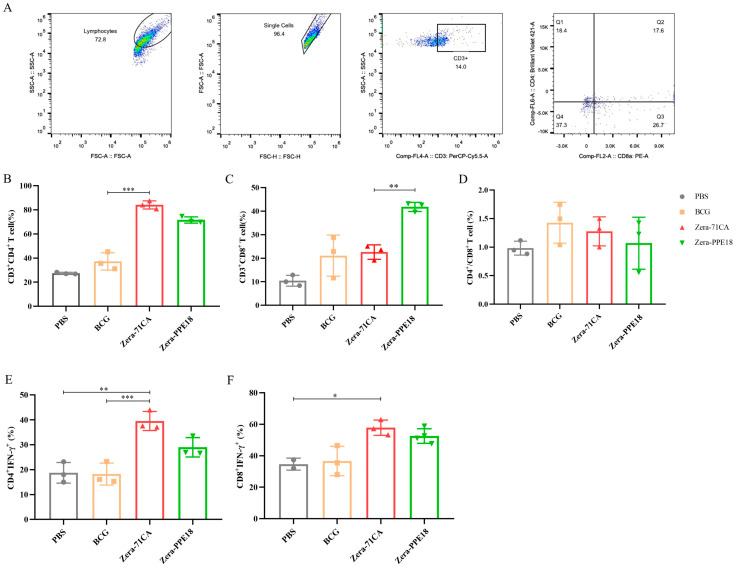
Nanoparticle-activated T cell immune response. Flow cytometer gate strategy (**A**); the percentages of CD3^+^CD4^+^ T cells (**B**), CD3^+^CD8^+^ T cells (**C**), CD4^+^/CD8^+^ (**D**), CD4^+^IFN-γ^+^ (**E**), and CD8^+^IFN-γ^+^ (**F**). T cells in the splenic lymphocytes of immunized mice. Data are expressed as the mean ± SD and were analyzed using one-way ANOVA with Bonferroni correction. ns denotes no significant difference; * *p* < 0.05; ** *p* < 0.01; *** *p* < 0.001 (n = 3).

CD8^+^ plays an important role in the clearance of tuberculosis foci, and CD4^+^/CD8^+^ reflects the state of immune balance of the organism. There were more CD3^+^CD4^+^ cells, CD3^+^CD8^+^ cells, and CD4^+^/CD8^+^ cells in the Zera-71CA/Zera-PPE18 group than in the control group. CD4^+^ cells can effectively promote the proliferation and differentiation of T lymphocytes and B lymphocytes, which are essential for effective cellular immunity. Activated T cells produce cytokines such as IFN-γ and TNF-α; IFN-γ can increase the activity of NK and CD8^+^ T cells and inhibit the spread of bacteria to the outside of the lungs (Figure 6A–F).

### 3.5. Security Testing

**Figure 7:**
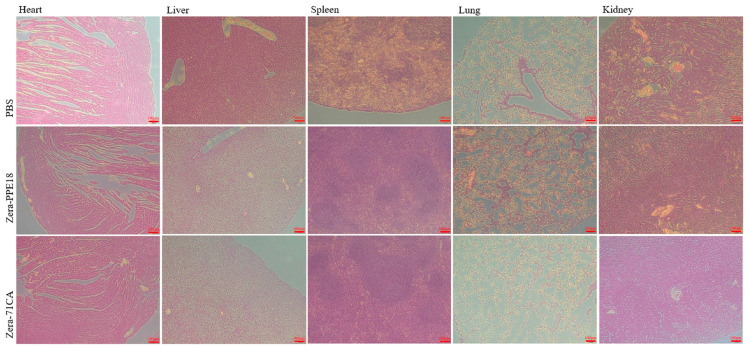
Tissue sections from the key organs were stained with hematoxylin and eosin (H&E).

After administering the Zera-71CA/Zera-PPE18 vaccine, no noticeable inflammatory responses or organ damage were observed in the major organs of mice, such as the heart, liver, spleen, lungs, and kidneys. Histological analysis showed that the tissue structure remained intact, with no evidence of cell necrosis, inflammatory infiltration, or abnormal cell growth. These results indicated that the vaccine is biocompatible and safe (Figure 7).

### 3.6. Zera-71CA/Zera-PPE18 Nanoparticle Induces Immune Response in Calves

**Figure 8:**
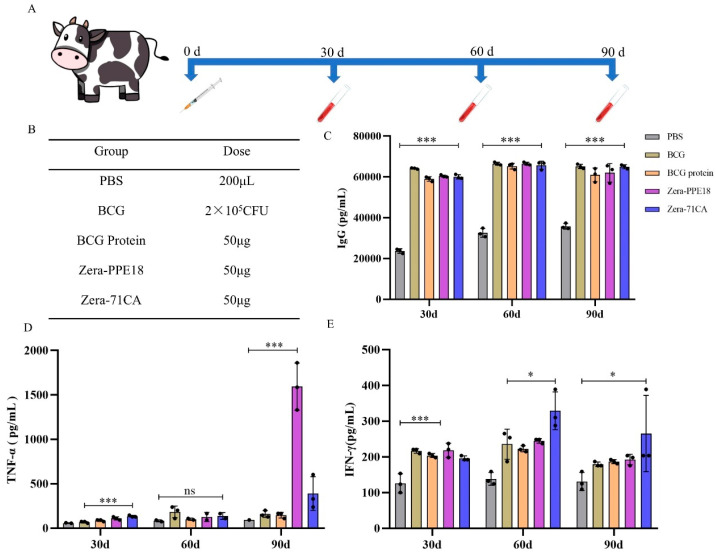
Evaluation of immunogenicity in calves. (**A**) Immunization protocol for calves; (**B**) determination of groups and doses; (**C**) analysis of IgG responses induced in immunized calves determined by indirect ELISA. * *p* < 0.05, *** *p* < 0.001, significant difference (one-way ANOVA with Bonferroni correction). Quantitative analysis of TNF-α (**D**) and TNF-γ (**E**) concentrations in serum of vaccinated calves. All analyses were performed in triplicate, and error bars show standard deviation (SD) (n = 3).

The immunogenic potential of the two nanoparticle vaccines was further tested in calves. At 30, 60, and 90 days after immunization (DAI), the serum IgG concentrations in the Zera-71CA/Zera-PPE18 group were significantly elevated (*p* < 0.001) compared to those in the control and commercial vaccine groups. Moreover, the levels of IFN-γ and TNF-α in the serum showed that at 90 DAI, calves immunized with the Zera-71CA nanoparticle vaccine had TNF-α and IFN-γ concentrations of 389.50 ± 189.74 pg/mL and 265.36 ± 106.87 pg/mL, respectively. In calves receiving the Zera-PPE18 vaccine, TNF-α and IFN-γ levels at 90 DAI were 1592.70 ± 264.40 pg/mL and 192.43 ± 14.84 pg/mL, respectively, significantly higher than those in the PBS, BCG, and BCG protein groups (*p* < 0.001; Figure 8A–E).

## 4. Discussion

At present, the BCG vaccine is the sole licensed vaccine for TB that is widely used. While the vaccine provides moderate protection for infants and children, it does not protect adolescents or adults, who are the major population affected by TB; therefore, the vaccine is not sufficient to curb the global TB epidemic [18,19], and there is a pressing demand for the creation of new and more potent tuberculosis vaccines.

The development of tuberculosis vaccines has evolved into a diversified landscape, encompassing live attenuated vaccines, subunit vaccines, viral vector-based vaccines, and vaccines utilizing nanoparticle delivery platforms. Among them, MTBVAC is one of the most representative live attenuated candidates. It is derived from a clinical isolate of human *M. tb* and attenuated through the deletion of two major virulence genes, phoP and fadD26, while retaining key immunodominant antigens such as ESAT-6 and CFP-10, which are absent in BCG. MTBVAC has completed Phase I and II clinical trials, demonstrating good safety and immunogenicity in both neonates and adults, and is currently undergoing Phase III clinical evaluation [20,21]. Another promising candidate is VPM1002, a recombinant BCG-based live vaccine. It enhances antigen release by inserting the listeriolysin gene and deletes the ureC gene (encoding ureidodiphosphoglucose dehydrogenase) to avoid intracellular acidification. VPM1002 is one of the most advanced and improved BCG vaccines, and it has already entered phase III clinical trials in several countries. In addition to live vaccines, subunit vaccines have gained considerable attention in recent years due to their excellent safety profile, flexible antigen design, and suitability for large-scale standardized production [22]. Notable examples include M72/AS01E, H56:IC31, and GamTBvac. M72/AS01E, co-developed by GSK and Aeras, contains a fusion antigen (M72) derived from the *M. tuberculosis* antigens Rv1196 and Rv0125, and is formulated with GSK’s proprietary AS01E adjuvant. In a Phase IIb trial, it demonstrated nearly 49.7% protective efficacy against active TB in latently infected adults, providing a strong foundation for the clinical potential of subunit vaccines [23,24]. H56:IC31, developed by the Statens Serum Institut in Denmark, combines three immunogenic antigens (Ag85B, ESAT-6, Rv2660c) and is adjuvanted with IC31, which consists of a cationic peptide and an oligonucleotide. This formulation significantly enhances the multifunctional CD4⁺ T-cell response and promotes long-lasting T-cell memory. H56:IC31 is currently in Phase II clinical trials [25,26]. Another candidate, GamTBvac, developed in Russia, utilizes a fusion of Ag85A and ESAT-6 antigens combined with an adjuvant, and has shown favorable immunogenicity in preclinical and early clinical studies [27]. It is currently undergoing further clinical evaluation. Emerging vaccine delivery technologies are also accelerating the evolution of TB vaccine platforms. Novel strategies involving nanoparticle delivery systems allow for precise antigen design, rapid development, and improved uptake and presentation by antigen-presenting cells. Researchers have explored fusing TB antigens with self-assembling nanostructures such as Zera, mi3, or ferritin to enhance immunogenicity, particularly to elicit strong cell-mediated immune responses essential for TB protection.

To improve the effectiveness of subunit vaccines, one strategy is to enhance immunogenicity by fusing the target protein with other proteins, often incorporating adjuvants into the vaccine formulation or adding signal sequences to facilitate protein assembly [28]. Proteins featuring repetitive sequence motifs, such as PBs, are more efficiently internalized by antigen-presenting cells, thereby strengthening the immune response. PBs are specialized organelles in maize seeds, derived from the endoplasmic reticulum (ER), where they store large quantities of maize proteins in a stable form. Once expressed and targeted to the ER for post-translational modifications, these alcohol-soluble maize protein peptides aggregate into large complexes, eventually self-assembling into PBs. The Zera sequence, a γ-maize alcohol protein with a proline-rich N-terminal, has been shown to play a crucial role in retaining proteins in the ER and facilitating PB formation in both maize seeds and a variety of eukaryotic cells [13,29]. Fusion of the HPV 16E7SH protein with Zera sequences enhanced the immune response, and in a mouse model, the protein vaccine successfully induced specific humoral and cellular immune responses and mediated tumor regression, indicating that the PBs formed by Zera induction have adjuvant activity [16]. Moreover, fusion of the HIV gp140 protein with the Zera sequence significantly increased protein immunogenicity and yield and led to high antibody titers [30]. Consequently, the Zera sequence offers a powerful and flexible approach for recombinant protein production by enabling their accumulation within organelles derived from the endoplasmic reticulum. The ability of Zera to induce PB formation holds significant promise for broad applications, particularly in the manufacturing of intracellular, non-secreted recombinant proteins [31].

In this research, we successfully employed the Zera-based nanoparticle platform to express the *M. tb* antigen PPE18. By tagging the protein with mCherry, we visualized nanoparticle expression via fluorescence under an inverted microscope (Figure 2), confirming localization at the plasma membrane in Sf9 cells (Figure 3) and the formation of protein bodies (PBs) (Figure 4). The presence of mCherry-labeled PPE18 within these PBs verified efficient expression, while subsequent mouse immunization experiments highlighted the vaccine’s strong immunogenicity. Specifically, mice vaccinated with Zera-71CA produced markedly higher levels of IgG antibodies on days 35 and 42 compared to PBS and Zera-PPE18 groups (Figure 5C), indicating robust humoral responses likely enhanced by the co-delivered MTB32C protein. Cellular immune responses were also significantly boosted, as shown by lymphocyte proliferation and flow cytometry analyses. The Zera-71CA/Zera-PPE18 vaccine activated both CD4^+^ and CD8^+^ T cells and led to elevated serum concentrations of IL-12p40 and TNF-α (Figure 5D–F), confirming its capacity to stimulate potent cellular immunity and favorable biocompatibility (Figure 6). Further evaluation in calves demonstrated that the Zera-71CA/Zera-PPE18 formulation induced IgG titers similar to those seen with live BCG and BCG protein vaccines, and significantly higher than those in the PBS group. It also increased IFN-γ and TNF-α levels, which are key markers of protective immunity against *M. tb* [32]. In the early phase post-vaccination, immune priming involving B and T cell activation and memory formation predominates, while cytokines such as TNF-α typically peak during the memory phase. Interestingly, TNF-α production in the Zera-PPE18 group was notably elevated only at day 90, suggesting a delayed but strong memory response and progressive T cell activation (Figure 8). However, the absence of a Zera-only control group in this study presented certain limitations. As Zera not only functions as a self-assembling domain but also possesses intrinsic immunostimulatory properties, the lack of such a control group limited mechanistic insights into Zera’s role in modulating immune activation and antigen presentation. In future studies, the pure Zera group should be included to clarify its independent contribution to immunogenicity and antigen processing. The release characteristics of the vaccine in vivo or in vitro should also be systematically investigated to further explore the release kinetics and immune response mechanism of the vaccine preparation and provide a theoretical basis for better prevention of TB infections.

## 5. Conclusions

This study presented the development and evaluation of Zera-based nanoparticles carrying the MTB32C-PPE18 fusion antigens. The findings demonstrated that the engineered nanoparticles were capable of eliciting both antibody-mediated and cellular immune responses in mice and calves, highlighting their promise as a vaccine candidate targeting PPE18 and MTB32C, as well as tuberculosis infection. Future research will involve testing this candidate in TB mouse models to assess its protective efficacy under both laboratory and practical conditions.

## Figures and Tables

**Table 1:** Antigens selected in this study.

Protein	NCBI Accession Number	Features/Functions	Selected Amino Acid Length
PPE18	NC_000962.3	PE/PPE protein family	391 aa
MTB32C	MTCI418B.07	Metabolism	132 aa

**Table 2:** Mouse vaccination strategy.

Group	Immunization Time Points	Dose
PBS	0, 14, 28	100 μL
BCG	0, 14, 28	5 × 10^4^ CFU
Zera-71CA nanoparticles	0, 14, 28	10 μg
Zera-PPE18 nanoparticles	0, 14, 28	10 μg

## Data Availability

The authors confirm that the data supporting the findings of this study are available within the article materials.

## Abbreviations

*M. tb*	*Mycobacterium tuberculosis*
TB	tuberculosis
PE	proline-glutamate
PPE	proline-proline-glutamate
BCG	Bacillus Calmette–Guérin
ER	endoplasmic reticulum
PBs	protein bodies
ELISA	enzyme-linked immunosorbent assay
SI	stimulation index
